# Supplementary material for: Voltage control of multiferroic magnon torque for reconfigurable logic-in-memory
Source: Nat Commun. 2024 Jul 16;15:5975. doi: 10.1038/s41467-024-50372-3 (PMC11252438; doi:10.1038/s41467-024-50372-3)
Supplement: Supplementary file 1 — Supplementary Information [file 41467_2024_50372_MOESM1_ESM.pdf]

Supplementary materials for

Voltage Control of Multiferroic Magnon Torque for Reconfigurable Logic-In-Memory

Yahong Chai*, Yuhan Liang*, Cancheng Xiao*, Yue Wang, Bo Li, Dingsong Jiang, Pratap Pal, Yongjian Tang, Hetian Chen, Yuejie Zhang, Hao Bai, Teng Xu, Wanjun Jiang, Witold Skowroński, Qinghua Zhang, Lin Gu, Jing Ma, Pu Yu, Jianshi Tang, Yuan-Hua Lin†, Di Yi†, Daniel C. Ralph, Chang-Beom Eom, Huaqiang Wu, Tianxiang Nan†

† Corresponding author. Email: linyh@tsinghua.edu.cn, diyi@mail.tsinghua.edu.cn, nantianxiang@mail.tsinghua.edu.cn

Table of Contents

Supplementary Notes……………………………………………………………….3

Supplementary Fig. 1…………………………………………………………...15

Supplementary Fig. 2…………………………………………………………...16

Supplementary Fig. 3…………………………………………………………...17

Supplementary Fig. 4…………………………………………………………...18

Supplementary Fig. 5…………………………………………………………...19

Supplementary Fig. 6…………………………………………………………...20

Supplementary Fig. 7…………………………………………………………...21

Supplementary Fig. 8…………………………………………………………...22

Supplementary Fig. 9…………………………………………………………...23

Supplementary Fig. 10..………………………………………………………...24

Supplementary Fig. 11…..……………………………………………………...25

Supplementary Fig. 12..………………………………………………………...26

Supplementary Fig. 13…..……………………………………………………...27

Supplementary Fig. 14…..……………………………………………………...28

Supplementary Fig. 15…..……………………………………………………...29

Supplementary References……………………………………………………...30

Supplementary Notes

Note 1. Model for voltage-controlled magnon torque in $BiFeO_3$ with two-variant stripe domain

We estimate the magnon transport in two-variant stripe domains with spin cycloid, in which spin cycloid propagates along $\vec{\boldsymbol{k}}$ orthogonal to ferroelectric polarization $\vec{\boldsymbol{P}}$ in $BiFeO_3$ (see Supplementary Fig. 1a). For $\vec{\boldsymbol{k}}$ in the x-y plane, the Néel order can be written as:

$$\vec{\boldsymbol{L}}(\mathbf{r}) = \vec{\boldsymbol{e}}_p \cos(\vec{\boldsymbol{k}}\cdot\vec{\boldsymbol{r}}) + \vec{\boldsymbol{e}}_k \sin(\vec{\boldsymbol{k}}\cdot\vec{\boldsymbol{r}}) \tag{1}$$

where $\vec{\boldsymbol{e}}_p$ and $\vec{\boldsymbol{e}}_k$ are the unit vectors of $\vec{\boldsymbol{P}}$ and $\vec{\boldsymbol{k}}$, and $|\mathbf{k}| = 2\pi/\lambda$ with λ being the cycloid characteristic wavelength. For domain 1, $\vec{\boldsymbol{e}}_p^1 = \vec{\boldsymbol{x}} cos\theta_p + \vec{\boldsymbol{z}} sin\theta_p$ and $\vec{\boldsymbol{e}}_k^1 = \vec{\boldsymbol{y}}$, corresponding to $\vec{\boldsymbol{k}}$ along $\vec{\boldsymbol{y}}$. For domain 2, $\vec{\boldsymbol{e}}_p^2 = -\vec{\boldsymbol{y}} cos\theta_p + \vec{\boldsymbol{z}} sin\theta_p$ and $\vec{\boldsymbol{e}}_k^2 = \vec{\boldsymbol{x}}$, corresponding to $\vec{\boldsymbol{k}}$ along $\vec{\boldsymbol{x}}$ (see Supplementary Fig. 1b). Here, $\theta_p = \arctan(1/\sqrt{2})$ and $\vec{\boldsymbol{x}}$, $\vec{\boldsymbol{y}}$ and $\vec{\boldsymbol{z}}$ are unit vectors. Since our sample is a thin $BiFeO_3$ film, we assume the Néel order distributes uniformly along $\vec{\boldsymbol{z}}$-direction. The averaged magnon current density propagates through the sample is given by[1-3]:

$$\vec{\boldsymbol{J}}^m = \frac{G_m}{\mathcal{A}} \int dxdy\ (\vec{\boldsymbol{\sigma}}\cdot\vec{\boldsymbol{L}})\,\vec{\boldsymbol{L}} \tag{2}$$

where G_m is magnon conductance per unit area, $\mathcal{A}$ is the area of x-y plane, and $\vec{\boldsymbol{\sigma}} = \vec{\boldsymbol{y}}$ is the spin polarization direction. The x-y plane is much greater than the cycloid period, i.e. $\mathcal{A} \gg \lambda^2$, thus Eq. (2) can be calculated in one period. By substituting the expression of Néel order of Eq. (1) into Eq. (2), we obtain the averaged magnon current density in two types of domains:

$$\vec{\boldsymbol{J}}_1^m = \frac{G_m}{2}\vec{\boldsymbol{e}}_k^1 \tag{3}$$

$$\vec{\boldsymbol{J}}_2^m = \frac{G_m}{2} cos\theta_p \vec{\boldsymbol{e}}_p^2 \tag{4}$$

As the magnon current is injected into ferromagnetic layer, the magnon torque density is given by[4]:

$$\vec{\boldsymbol{\tau}}_m = \vec{\boldsymbol{M}} \times (\vec{\boldsymbol{J}}^m \times \vec{\boldsymbol{M}}) \tag{5}$$

For PtCo, the magnetization $\vec{\boldsymbol{M}}$ is along $\vec{\boldsymbol{z}}$-direction, thus we obtain the magnon torque density as:

$$\tau_m^1 = G_m/2 \tag{6}$$

$$\tau_m^2 = \frac{G_m}{2} cos^2\theta_p \tag{7}$$

From the expression of Equation (6) and Equation (7) above, we find that for injected spin polarization $\vec{\boldsymbol{\sigma}}$ along $\vec{\boldsymbol{y}}$, the magnon transport is more efficient through domain 1, leading to a larger magnon torque on magnetization.

In rhombohedral $BiFeO_3$, the spin cycloid direction ($\vec{\boldsymbol{k}}$ in Supplementary Fig. 1c, purple arrows) is orthogonal to the in-plane component of ferroelectric polarization (bold yellow arrows)[5]. Therefore, the magnon transport through $BiFeO_3$ is expected to be quite different between the two domains, depending on the relative angle between spin polarization of spin current and the $\vec{\boldsymbol{k}}$. In [001] direction $BiFeO_3$ with two-variant domain structure, the ferroelectric polarization is along [111] with both in-plane and out-of-plane components. Therefore, the application of gate voltage also affects the in-plane component. Indeed, as shown in Supplementary Fig. 1c, after applying the V_G, the relative population of the two domains are modulated[6]. This modulation could lead to the change of magnon transport behaviors.

Note 2. Structure characterization of the $SrRuO_3/BiFeO_3$/PtCo and ferroelectricity of $BiFeO_3$ thin film

We first confirmed the high crystal quality of epitaxial $SrRuO_3/BiFeO_3$ heterostructure using X-ray diffraction, showing clear $(200)_{pc}$ Bragg peaks of $BiFeO_3$ and $SrRuO_3$ layers (see Supplementary Fig. 2a). The surface morphology of our $DyScO_3$/11 nm $SrRuO_3$/120 nm $BiFeO_3$ sample shows an atomically flat surface with the step-and-terrace structure and surface roughness better than 0.3 nm (see Supplementary Fig. 2b). The as-grown $SrRuO_3/BiFeO_3$ heterostructure shows ferroelectric two-variant stripe domain, revealed by in-plane PFM phase image (see Supplementary Fig. 2c). To investigate the polarization switching behavior, bias voltages of -5 V was applied to $BiFeO_3$ layer through the PFM tip with the $SrRuO_3$ layer grounded, as shown in Supplementary Fig. 2d. The out-of-plane component of ferroelectric polarization is switched from initially downward to upward (the right of Supplementary Fig. 2d), while the in-plane PFM shows the change of domain population after applying bias voltage (the left of Supplementary Fig. 2d).

Electron energy loss spectroscopy (EELS) mapping of elements distribution in an 11 nm $SrRuO_3$/50 nm $BiFeO_3$/PtCo sample is shown in Supplementary Fig. 2e, identifying the sharpness of $SrRuO_3$/$BiFeO_3$ and $BiFeO_3$/PtCo interfaces.

Note 3. Additional experimental results of magnon-torque-induced switching in $SrRuO_3$/$BiFeO_3$/PtCo and the current-induced self-switching in PtCo

The structure of the 11 nm $SrRuO_3$/120 nm $BiFeO_3$/PtCo sample for magnon-torque-induced switching is shown in Supplementary Fig. 4a. The additional results of magnon-torque-induced switching of magnetization under various in-plane magnetic field H_x for Fig. 2 are displayed in Supplementary Fig. 4b. The structure of the control sample (PtCo on Si substrate) is shown in Supplementary Fig. 4c, and corresponding anomalous Hall resistance loop as sweeping out-of-plane magnetic field H_z is shown in Supplementary Fig. 4d. The current-induced-switching results under $\mu_0 H_x$ up to ± 50 mT for control sample is shown in Supplementary Fig. 4e, and no self-switching effect[7] is observed within the same applied current range of that in $SrRuO_3$/$BiFeO_3$/PtCo sample (Supplementary Fig. 4b). Since all current flows into the PtCo layer, the current density in PtCo layer is much larger than that in the $SrRuO_3$/ $BiFeO_3$/PtCo sample. The Hall bar size of PtCo sample is same with that of the $SrRuO_3$/ $BiFeO_3$/PtCo sample. To further rule out the self-switching torque arising due to the $BiFeO_3$/PtCo interface, we fabricated $DyScO_3$/120 nm $BiFeO_3$/PtCo sample (without $SrRuO_3$) and conducted current switching experiments, see schematic in Supplementary Fig. 4f. As shown in Supplementary Fig. 4g, no magnetization switching can be observed, validating our conclusion.

Note 4. The reproducibility of threshold current

To check the reproducibility of I_c in each device, we have also carried out the current switching measurements for multiple times. Supplementary Figure 5 shows the I_c for one representative device across ten successive measurements, revealing that the variation of I_c is smaller than 2%. The error bar of Ic is determined by the standard deviation of these measurements. It's important to note that the modulation of Ic by voltage (~14%) is much larger than the variation of I_c.

Note 5. Determination of threshold switching current density

For Hall device, the current density is calculated according to parallel resistance model. In our control samples, the resistivity of PtCo layer on Si/SiO_2 substrate ρ_{PtCo} is estimated to be about 120 μΩ·cm, and the resistivity of 11 nm $SrRuO_3$ layer on $DyScO_3$ substrate ρ_{SRO} is about 325 μΩ·cm. The current density in $SrRuO_3$ layer can be expressed as:

$$j_{SRO} = \frac{I_w}{W} \times \frac{\rho_{PtCo}}{\rho_{SRO} \cdot d_{PtCo} + \rho_{PtCo} \cdot d_{SRO}} \quad (8)$$

where the I_w is the magnitude of current pulse, W = 16 μm is the width of device, t_{PtCo} = 8.3 nm is the thickness of PtCo layer, and d_{SRO}= 11 nm is the thickness of SRO layer. In Figure 2, the threshold current I_c = 16 mA, corresponding to $j_{SRO} \approx 3.0 \times 10^6$ A/cm^2.

In Figure 3 we show the current switching of PtCo in pillar devices. The current density is calculated as $j_{SRO} = \frac{I_w}{W \cdot d_{SRO}} \approx 2.4\times 10^6$ A/cm^2 (I_c = 8 mA, W = 30 μm) because there is no current shunting in PtCo layer. We find the critical current densities in the Hall bar (Figure 2) and pillar devices (Figure 3) are consistent.

Note 6. Experimental results of ST-FMR measurements

The ST-FMR measurements were carried out at room temperature. During ST-FMR measurements, a microwave current I_{rf} at a fixed frequency was applied with the in-plane magnetic field swept from 0 to 0.25 T to drive the resonance of the ferromagnetic layer NiFe. The representative optical image of the device for the ST-FMR measurement is shown in Supplementary Fig. 6a. The amplitude of the microwave current is modulated at a low frequency (1.713 kHz), and the mixing voltage V_{mix} is detected through a lock-in amplifier. The resonance spectra were obtained at a fixed frequency with sweeping an in-plane external magnetic field H_{ext}. Typical ST-FMR results of various microwave frequencies for 11 nm $SrRuO_3$/120 nm $BiFeO_3$/5 nm NiFe are shown in Supplementary Fig. 6b. The device resistance as a function of magnetic field angle φ due to the anisotropic magnetoresistance (AMR) of NiFe is shown in Supplementary Fig. 6c. The ST-FMR V_{mix} is a combination of I_{rf} and AMR, which can be then written in the form as[8]:

$$V_{mix} = V_S \frac{W^2}{(\mu_0 H_{ext} - \mu_0 H_{FMR})^2 + W^2} + V_A \frac{W(\mu_0 H_{ext} - \mu_0 H_{FMR})}{(\mu_0 H_{ext} - \mu_0 H_{FMR})^2 + W^2} \tag{9}$$

where W is the half-width-at-half-maximum resonance linewidth, and H_{FMR} is the resonance field. V_S and V_A are the symmetric and antisymmetric amplitude of the Lorentzian, and can be expressed as[9]:

$$V_S = -\frac{I_{rf}}{2}(\frac{dR}{d\varphi})\frac{1}{\alpha(2\mu_0 H_{FMR} + \mu_0 M_{eff})}\tau_{m,DL} \tag{10}$$

$$V_A = -\frac{I_{rf}}{2}(\frac{dR}{d\varphi})\frac{\sqrt{1 + M_{eff}/H_{FMR}}}{\alpha(2\mu_0 H_{FMR} + \mu_0 M_{eff})}\tau_{m,FL} \tag{11}$$

where I_{rf} is the microwave current, R(φ) is the device resistance as a function of angle φ of in-plane magnetic field based on the AMR, and $\mu_0 M_{eff}$ is the effective magnetization of NiFe and α is the Gilbert damping. Typical ST-FMR spectrum at 7 GHz with φ=130° is shown in Supplementary Fig. 6d. The symmetric and antisymmetric components can be obtained by fitting the spectrum to symmetric and antisymmetric Lorentzian functions according to Eq. (9) to Eq. (11). Moreover, the magnetic field angle φ dependence of V_S and V_A components both follow $sin2\varphi\cos\varphi$ dependence (see Supplementary Fig. 6e), showing the magnon current carries spin polarization along $\vec{\boldsymbol{y}}$.

The magnitude of magnon torque efficiency can be determined by extracting the symmetric and antisymmetric amplitude from Eq. (10) and Eq. (11), expressed as[10]:

$$\xi_{m,DL} = \frac{V_S}{V_A}\frac{e\mu_0 M_s ht}{\hbar}\sqrt{1 + \frac{M_{eff}}{H_0}} \tag{12}$$

where M_s and t are the saturation magnetization and the thickness of NiFe, h is the thickness of $SrRuO_3$ layer, $\hbar$ is the reduced Planck's constant, e is the electron charge, H_0 is the resonance field. The effective magnetization M_{eff} is obtained by measuring the frequency dependent H_{FMR} (see Supplementary Fig. 6f), with a fit to Kittel equation expressed as[9]:

$$2\pi f = \gamma\sqrt{\mu_0 H_{res}(\mu_0 H_{res} + \mu_0 M_{eff})} \tag{13}$$

where $\mu_0 H_k$ is the in-plane magnetic anisotropy field and γ is the gyromagnetic ratio. We find $\xi_{m,DL}$ = 0.012 at room temperature for the 11 nm $SrRuO_3$/120 nm $BiFeO_3$/5 nm NiFe via ST-FMR measurements.

Note 7. Experimental results of SHHV measurements

The SHHV measurements were conducted on the 11 nm $SrRuO_3$/120 nm $BiFeO_3$/5 nm NiFe. By applying an ac current I_{ac}, the magnetization can be oscillated by field-like magnon torque $\tau_{m,FL}$ and damping-like magnon torque $\tau_{m,DL}$ around the equilibrium position, resulting in second harmonic hall voltage $V_{2\omega}$, as shown in Supplementary Fig. 7a. The $V_{2\omega}$ is measured while an external magnetic field H_{ext} is rotated within the sample plane, to different angles φ with respect to I_{ac}. The measured $V_{2\omega}$ is fitted against φ using[11,12]:

$$V_{2\omega} = I_{ac}\frac{R_{PHE}\tau_{m,FL}}{\gamma\mu_0 H_{ext}}cos(2\varphi)cos\varphi + I_{ac}[\frac{R_{AHE}\tau_{m,DL}}{2\gamma(\mu_0 M_s + \mu_0 H_{ext})} + R_{ANE}]cos\varphi + I_{ac}R_x sin(2\varphi) \quad (14)$$

where R_{PHE}, R_{AHE}, and γ are the planar Hall resistance, the anomalous Hall resistance, and the gyromagnetic ratio, respectively. The $cos(2\varphi)cos\varphi$ term describes the strength of $\tau_{m,FL}$. The $cos\varphi$ term consists of contributions from both the $\tau_{m,DL}$ and the anomalous Nernst effect (V_{ANE}). The $sin2\varphi$ term is proportional to resistance R_x, and plays a minor role in the $V_{2\omega}$[12]. The typical $V_{2\omega}$ result is shown in Supplementary Fig. 7b, which can be well fitted using Eq. (14). We note that, the R_{PHE} was obtained by monitoring first harmonic voltage $V_{1\omega}$ as rotating H_{ext} within the sample plane (see Supplementary Fig. 7c) and the $V_{2\omega}$ spectrum with various magnitude of H_{ext} were also detected (see Supplementary Fig. 7d). Then, we plot $I_{ac}\frac{R_{PHE}\tau_{m,FL}}{\gamma H_{ext}}$ against $\frac{1}{\mu_0 H_{ext}}$ to extract the $\tau_{m,FL}$ from the gradient of the linear fit (see Supplementary Fig. 7e). After removing the constants from the amplitude of the $cos\varphi$ term, we plot $I_{ac}\frac{R_{AHE}\tau_{m,DL}}{2\gamma(\mu_0 M_s+H_{ext})}$ against $\frac{1}{(\mu_0 M_s+\mu_0 H_{ext})}$ to extract the $\tau_{m,DL}$ from the slope of the linear fit (see Supplementary Fig. 7f). The efficiency of damping-like magnon torque is estimated using $\xi_{m,DL} = \frac{M_s t_{FM}}{\hbar/2e}\cdot\frac{\tau_{m,DL}}{j_c}$, where j_c is the current density in the $SrRuO_3$ layer (about 1.1×10^6 A/cm^2), and t_{FM} is the thickness of the NiFe layer. We find $\xi_{m,DL}$ = 0.027 at room temperature for the 11 nm $SrRuO_3$/120 nm $BiFeO_3$/5 nm NiFe via SHHV measurements.

Note 8. Magnon torque efficiency in $SrRuO_3$/$BiFeO_3$/PtCo samples

We conducted SHHV measurements for a 11 nm $SrRuO_3$/120 nm $BiFeO_3$/PtCo tri-layer. The measurement geometry is shown in Supplementary Fig. 8a, as an ac current I_{AC} is applied along the x-direction, and an alternating magnon current is injected into PtCo layer, exerting a damping-like magnon torque effective field ($H_{m,DL}$) to induce the oscillation of PtCo magnetization. As the in-plane magnetic field H_x larger than anisotropic field H_K, the magnetization vector M is aligned along the x-direction, leading to the SHH resistance to be written as[13,14]:

$$R_{xy}^{2\omega}=\frac{R_{AHE}}{2}\frac{H_{m,DL}}{|H_x|-H_K}+R_{PHE}\frac{H_{FL}}{|H_x|}+R_{thermal} \quad (15)$$

Where R_{AHE} and R_{PHE} are the AHE and planar Hall resistances and $R_{thermal}$ is the thermal contribution from anomalous Nernst and spin Seebeck effects. Since $R_{PHE} << R_{AHE}$, the second term can be neglected. Thus, we can estimate the effective field H_{DL} via fitting the $R_{xy}^{2\omega}$ data in the large in-plane field regime.

We then apply I_{AC} along x direction with in-plane field parallel to the current. The results for first and second harmonic hall resistance are shown in Supplementary Fig. 8b and Fig. 8c. By changing the amplitude of current density (J) and fitting the second harmonic resistance, as shown in Supplementary Fig. 8d below, the $H_{m,DL}$/J is estimated to be 0.077 mT per 10^6 A/cm^2. The damping-like spin-torque efficiency can be estimated as $\xi_{DL}=\frac{2e}{\hbar}M_s t_{Co}\frac{H_{DL}}{J_e}$. Here, we measure the saturated magnetization of Co as M_s = 1100 emu/cm^3 consistent with reported values[15], see Supplementary Fig. 3, and t_{Co} = 1.6 nm for the thickness of Co. The damping-like torque efficiency $\xi_{m,DL}$ is calculated to be around 0.041, which is within the same order of magnitude with the results measured by ST-FMR ($\xi_{m,DL}$=0.012) and SHHV ($\xi_{m,DL}$=0.027) in $SrRuO_3$/$BiFeO_3$/NiFe tri-layer.

Note 9. $BiFeO_3$ thickness dependence of magnon transport

Supplementary Figure 9 shows the damping-like magnon torque efficiency $\xi_{m,DL}$ as a function of BFO thickness, indicating a non-monotonic trend similar to the NiO-based heterostructures[16]. In Supplementary Fig. 10, we observe the magnon-torque-induced magnetization switching with the critical switching current density of about 2.8×10^6 A/cm^2. This is slightly smaller than that of the 11 nm $SrRuO_3$/120 nm $BiFeO_3$/PtCo sample (Fig. 2).

Note 10. Leakage current in the $BiFeO_3$/PtCo pillar

To rule out the contribution of spin transport mediated by electrons, we conducted leakage current measurement in $BiFeO_3$ thin films. In the same device shown in Fig. 3 (25-μm-diameter 11 nm $SrRuO_3$/120 nm $BiFeO_3$/PtCo circular micro-pillar), we measured the current-voltage (I-V) characteristic curve using Keithley 4200A-SCS Parameter Analyzer. The leakage current is estimated to be about 10^{-8} A at 1 V (see Supplementary Fig. 11), corresponding to a junction resistance of about 10^8 Ω and a resistivity about 4.1×10^7 Ω·cm for the 120 nm $BiFeO_3$ film, which indicates our 120 nm $BiFeO_3$ films are good insulators. Therefore, we rule out the contribution from the electron mediated spin transport.

Note 11. Experimental results of polar MOKE measurements for the voltage-controlled magnetic anisotropy effect and discussion on piezoelectrical strain effect.

To exclude the variation of perpendicular magnetic anisotropy due to voltage-controlled magnetic anisotropy effect[17], we measured the out-of-plane polar MOKE signals as a function of out-of-plane magnetic field H_z for the 11 nm $SrRuO_3$/120 nm $BiFeO_3$/PtCo sample before and after applying V_G at the same position of the pillar (as shown in Fig. 3). The polar MOKE hysteresis loops are shown in Supplementary Fig. 12, and we observed negligible changes in the hysteresis loop of the cell before and after V_G application. In our system, the PMA in PtCo layer is very robust and show negligible modulation of coercive field (H_c remains at around 20 mT before and after applying voltage) and ferromagnetic remanence (M_r/M_s = 0.97 and 0.98 before and after applying voltage) in the two-variant domain pillar devices (see Supplementary Fig. 12), which excludes the voltage-controlled magnetic anisotropy effect for the magnon torque switching.

The piezoelectric strain can be induced in ferroelectric materials such as in $BiFeO_3$[18]. However, we believe that strain does not play a major role in our devices for the following reasons. First, the amount of strain induced in our samples should be negligible because of the substrate clamping effect. Moreover, the modulation of magnon transport behavior is observed in the remnant states after removing the voltage. Considering the domain architectures and 180° ferroelectric polarization switch in each domain, the remnant strain is expected to be almost the same. Furthermore, even if

there is a finite remnant strain in $BiFeO_3$, the magnetic anisotropy is sensitive to the strain and the change of magnetic anisotropy can serve as a probe for the existence of strain. In Supplementary Fig. 12, no clear change of the magnetic anisotropy in PtCo is observed before and after applying gate voltages. This indicates a negligible strain effect.

Note 12. Definitions of IN and settings of OUT_{i-1} for performing all 16 Boolean logic functions

The charge current pulse I_w with different direction and amplitude ($-I_{c2}<I_w<-I_{c1}$, $-I_{c1}<I_w<I_{c1}$ or $I_{c1}<I_w<I_{c2}$), polarized state ("↓" or "↑") and magnetization state ("↓" or "↑") represent IN, W (Weight) and OUT_i/OUT_{i-1}, respectively. W can be switched by voltage pulse. OUT_{i-1} is the state variable of ferromagnetic layer which should be initialize "↓" ($I_w>I_{c2}$) or "↑" ($I_w<-I_{c2}$) dependent on the requirements of different logic and IN. The logic operation can be performed with different current pulse I_w. Hence, the definitions of IN and initialization of OUT_{i-1} for performing different Boolean logic functions can be ascertained by logical derivation and the lookup of truth table. For the most Boolean logic functions, searching directly in truth table is an easier way to complete the ascertainment. In another way, the logic equation, reflecting the writing behavior of device, can be derivation with simplification with Karnaugh map as:

$$\mathrm{OUT_i} = \bar{S} \cdot OUT_{i-1} + \bar{A} \cdot OUT_{i-1} + \bar{W} \cdot OUT_{i-1} + \bar{S} \cdot A \cdot W \quad (16)$$

where the S and A are the symbol ("1" for positive and "0" for negative) and amplitude ("1" for $|I_{c1}|<|I_w|<|I_{c2}|$ and "0" for $0<|I_w|<|I_{c1}|$) of I_w, respectively. The combination of S and A has four possible values, where "00" and "10" are equivalent. The definition of IN and initialization of OUT_{i-1} can be clarified by presetting OUT_{i-1} and constructing the objective function (see Supplementary Fig. 13).

Note 13. Reversible voltage control of magnon-torque-induced switching in a sample with the mono-domain $BiFeO_3$

To realize reversible voltage-control magnon torque, we fabricate 15 nm $SrRuO_3$/150 nm $BiFeO_3$/PtCo on (100) $SrTiO_3$ single-crystal substrates with 4° miscut toward $[110]_{pc}$ direction. Due

to the epitaxy strain and 4° miscut of $SrTiO_3$ substrate, the $BiFeO_3$ layer shows ferroelectric mono-domain structure, revealed by PFM phase images[17] (Supplementary Fig. 14a and Supplementary Fig. 14b). The out-of-plane PFM phase signal hysteresis loop is monitored by sweeping out-of-plane applied voltage, indicating the coercive voltage about 10 V to switch the ferroelectric polarization, as shown in Supplementary Fig. 14c. Then, *in-situ* voltage-controlled magnon-torque-induced switching of magnetization was conducted. The I_w was applied to spin-current channel after applying V_G to PtCo/$BiFeO_3$ pillar, with probe tip keeping contact with pillar (see Materials and Methods for the device fabrication). An external magnetic field was applied during the magnon-torque-induced switching. The pulse width of I_w is 1 ms. We found that I_{th} =12.0±0.1 mA (12.5± 0.2 mA) after applied V_G = +20 V (-20 V) for several cycles, as shown in Supplementary Fig. 14d. The corresponding polar MOKE images for magnon-torque-induced switching with various I_w after applying V_G are displayed in Supplementary Fig. 14e.

We observed a clear modulation of I_c, albeit with a smaller ratio compared to the two-domain case. This discrepancy can be understood as a different domain structure and spin cycloid configuration in $BiFeO_3$ due to the confinements of strain and miscut of substrate. The mono-domain $SrTiO_3$/$SrRuO_3$/$BiFeO_3$/PtCo sample presents a singular spin cycloid direction. Upon ferroelectric switching, the cycloid plane can be slightly modulated as previously demonstrated[17]. The relatively modest modulation ratio of Ic in the mono-domain sample likely stems from the change in the cycloid plane following ferroelectric switching. We also conduct voltage control magnon torque switching experiments for multiple devices, finding modulation ability about 10%~14% in two-variant samples and 2%~4% mono-domain samples, respectively. We speculate that the ferroelectric domain structure would strongly affect I_{c1} and I_{c2}. These findings underscore the potential for achieving a higher modulation ratio through deliberate engineering of the antiferromagnetic structure, an avenue that warrants further investigation.

Note 14. MMST-MTJs crossbar array and the equivalent circuits

Built on the MMST device in the main text, we can demonstrate a MMST-MTJs crossbar array for high throughput multiply–accumulate (MAC) operations. The schematic is shown in

Supplementary Fig. 15a, which consists of n MMST-MTJ cells stacked on a write bit line for a shared write current. Each cell is controlled by a gate voltage through transistor. When the multiplier and accumulation operations are performed, the results of each basic operation are obtained via the reconfigurable characteristics of the MMST device and stored in the MTJ. Then the same voltage is applied to each source line. In this way, the reading line accumulates the conductance of each MTJ on the same column to obtain the total current, which is converted to the final digital result using an analog-to-digital converter (ADC). The equivalent circuit of a MMST-MTJs column for MAC operation is also shown in Supplementary Fig. 15b. Two non-volatile variable states of ferroelectric polarization and conductance of MTJ are equivalent to two registers in each MMST-MTJ that are utilized to store the synaptic weight and intermediate calculation result, respectively. Different types of logic operations that can be implemented in the MMST device (as shown in the Supplementary Fig. 7) is equivalent to the reconfigurable gate. The intermediate calculation results are accumulated via the ADC which is equivalent to an adder.

Note 15. Comparison of different spintronic devices

From a memory device perspective, the integration of ferroelectric polarization and magnetization offers no distinct advantages over a system with two magnetic bits. However, from the perspective of in-memory computing, the non-volatile modulation of magnetization provided by ferroelectric polarization (whereas VCMA is a volatile modulation) empowers a single device to perform all 16 Boolean logic operations within two steps, which cannot be achieved by a system with two magnetic states[19]. This has the potential to broaden the computational capabilities of crossbars based on emerging non-volatile memories. Meanwhile, more complex logical functions can be further realized based on the simple Boolean logic operations, which also elevates the edge of MRAM in digital in-memory computing. Furthermore, we have summarized the device properties and functionalities between our proposed in-memory logic device and other spin-based devices in Table 1. We also envision great potential for such non-volatile control in future neuromorphic computing.

Supplementary Table 1. Comparison of different spintronic devices

Device	Write information	Gate controllability	Logic function	Reference
Spin-transfer torque MRAM	Spin-transfer torque	N/A	N/A	Nature 601, 211–216 (2022)
Spin-orbit torque MRAM	Spin-orbit torque	N/A	N/A	Science 336, 555–558 (2012)
Voltage-controlled spin–orbit torque	Spin-orbit torque	Voltage-control of magnetic anisotropy, volatile	XNOR logic (with two devices)	Nat. Electron. 1, 398–403 (2018)
Magnetoelectric spin–orbit logic (proposed)	Magnetoelectric control of magnetic vector	N/A	Buffer logic (with single device)	Nature 565, 35–42 (2019)
Multiferroic magnon spin-torque device	Magnon-mediated spin torque	Magnetoelectric control of magnon transport, non-volatile	Reconfigurable 16 Boolean logics (with single device)	This work

Supplementary Figures

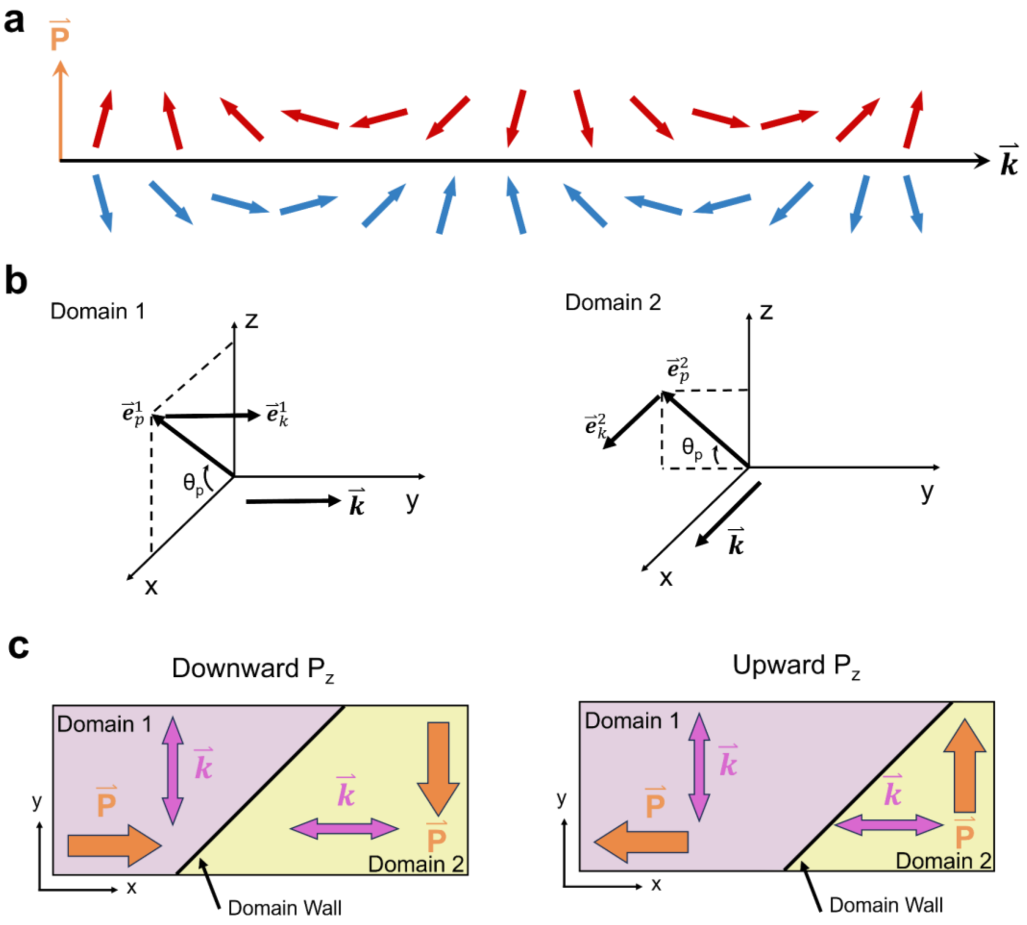

Supplementary Figure 1. Schematic illustration of the model for voltage-controlled magnon torque in $BiFeO_3$ with two-variant stripe domain structure. a, Schematic of spin cycloid structure in $BiFeO_3$ on $DyScO_3$. The red and blue arrows represent the two sublattice magnetic moments. **b**, Néel order in domain 1 and domain 2. The cycloid propagation direction $\vec{k}$ of each domain is denoted. **c**, Schematic top-view of ferroelectric domain structure change before and after applying V_G. Purple region for domain 1 and yellow region for domain 2. Purple arrows represent the cycloid propagation direction $\vec{k}$. Bold yellow arrows represent in-plane ferroelectric polarization component.

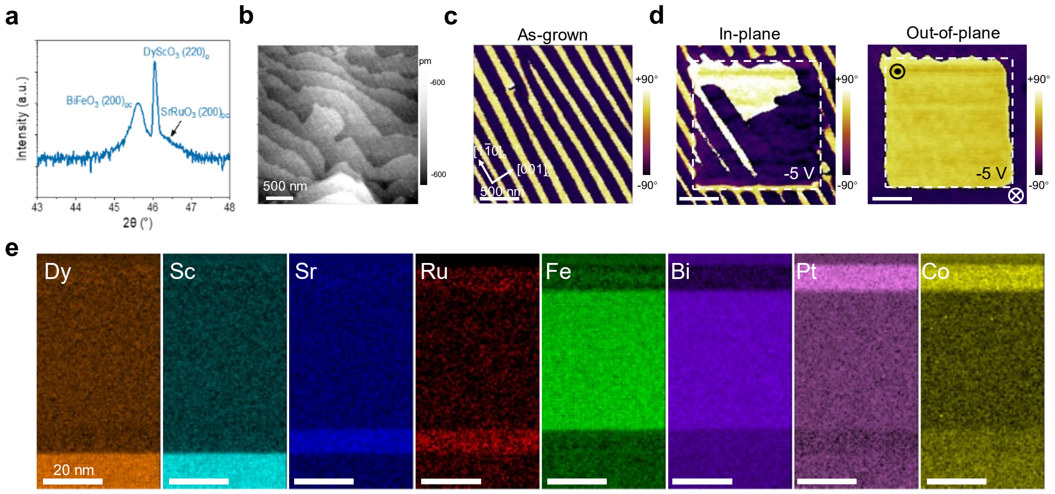

Supplementary Figure 2. Structure characterization of the $SrRuO_3$/$BiFeO_3$/PtCo and ferroelectricity of $BiFeO_3$ thin film. a, 2θ-ω x-ray diffraction scan of $SrRuO_3$/$BiFeO_3$/PtCo heterostructure on $(110)_o$ $DyScO_3$ substrate zoomed along the $(220)_o$ peak of $DyScO_3$. **b**, the surface morphology of fabricated $SrRuO_3$/$BiFeO_3$ revealed by AFM. The scale bar is 500 nm. **c**, In-plane PFM phase image of the $SrRuO_3$/$BiFeO_3$ sample taken at the as-grown state, showing two-variant stripe domain structure. The scale bar is 500 nm. **d**, In-plane (left) and out-of-plane (right) PFM phase images of the $SrRuO_3$/$BiFeO_3$ sample taken after the switching written by negative bias voltage of -5 V (area squared by white dashed line). The scale bar is 500 nm. **e**, Electron energy loss spectroscopy (EELS) mapping of elements distribution in an 11 nm $SrRuO_3$/50 nm $BiFeO_3$/PtCo sample. The scale bar is 20 nm.

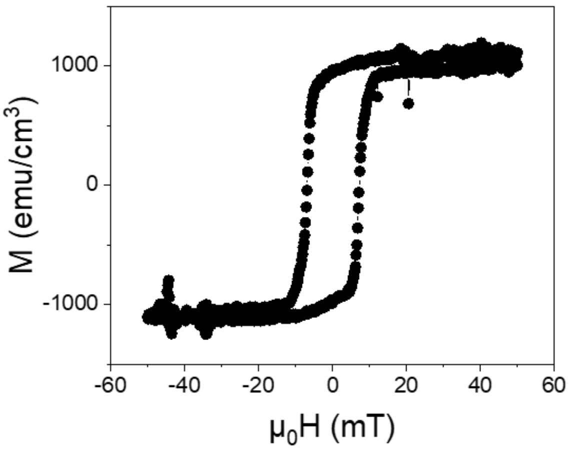

Supplementary Figure 3. The out-of-plane magnetization hysteresis loop measurement of a 11 nm $SrRuO_3$/ 120 nm $BiFeO_3$/ Pt(2)/[Co(0.4)/Pt(0.92)]×3/Co(0.4)/Pt(2) sample. Paramagnetic background from $DyScO_3$ substrate has been removed.

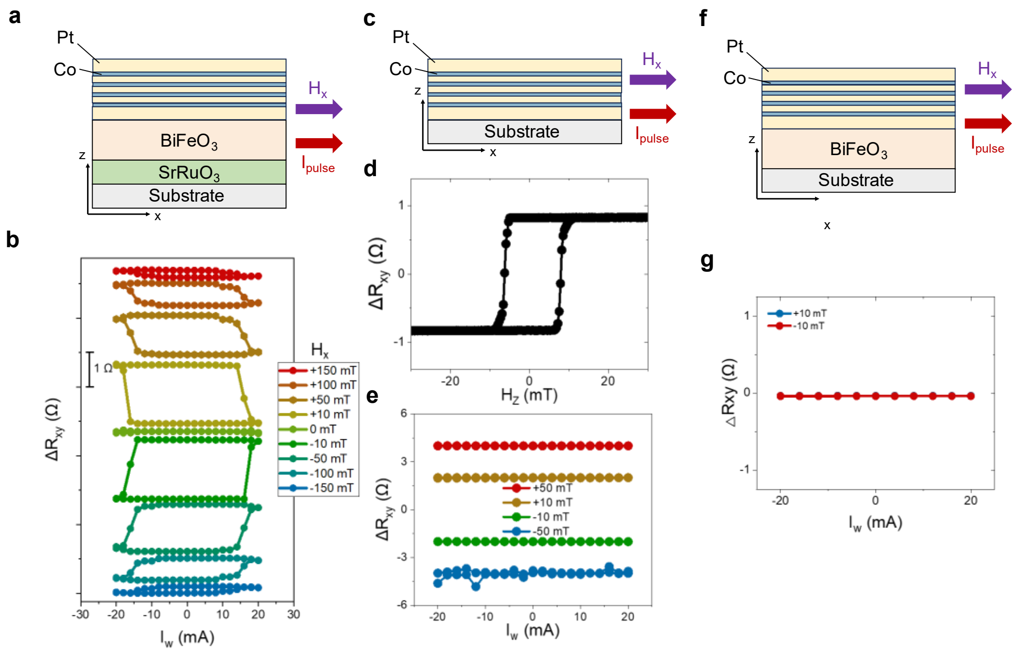

Supplementary Figure 4. Additional experimental results of magnon-torque-induced switching in $SrRuO_3/BiFeO_3$/PtCo and the current-induced self-switching in PtCo. a, Schematic of the layered structure for the 11 nm $SrRuO_3$/120 nm $BiFeO_3$/PtCo. **b**, Magnon-torque-induced switching of magnetization under various in-plane magnetic field H_x for the 11 nm $SrRuO_3$/120 nm $BiFeO_3$/PtCo. The anomalous Hall resistance ΔR_{xy}-I_w loops are manually shifted for better visualization. **c**, Schematic of the layered structure for the PtCo on a Si substrate. **d**, Anomalous Hall resistance loop for PtCo as sweeping out-of-plane magnetic field H_z. **e**, The anomalous Hall resistance ΔR_{xy}-I_w loops for PtCo under various $\mu_0 H_x$. **f**, Schematic of the layered structure for the PtCo on $BiFeO_3$ thin film without $SrRuO_3$. **g**, The anomalous Hall resistance ΔR_{xy}-I_w loops for PtCo on $BiFeO_3$.

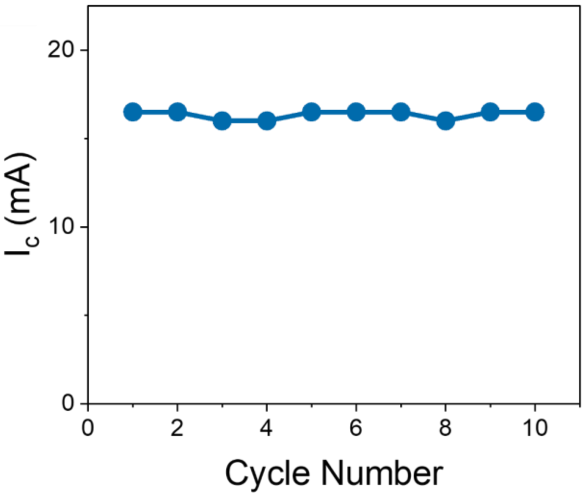

Supplementary Figure 5. Variation of threshold current I_c for magnon-torque-induced magnetization switching of the 11 nm $SrRuO_3$/120 nm $BiFeO_3$/PtCo samples in ten successive switching.

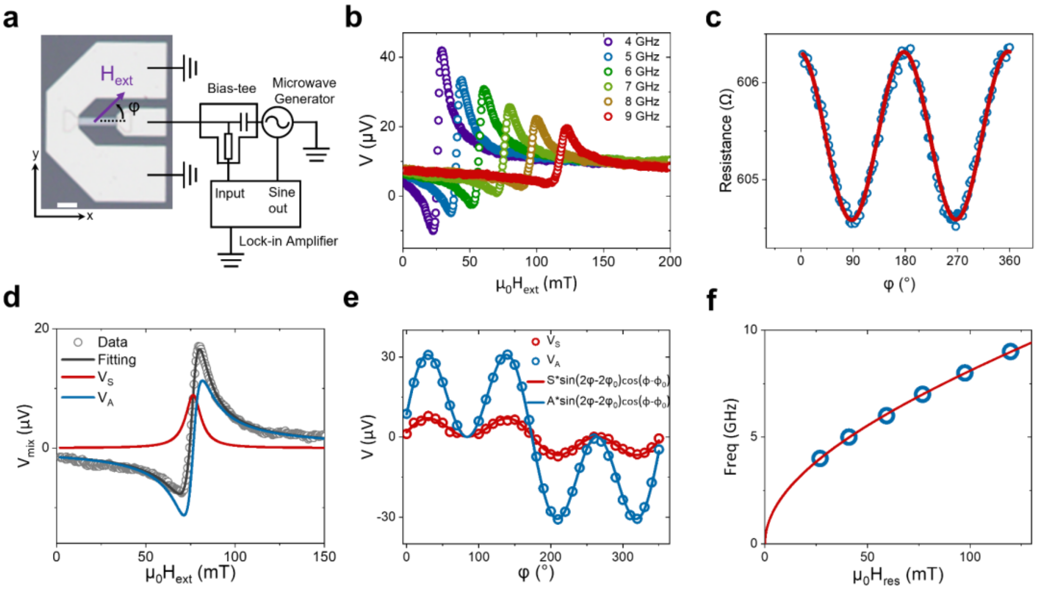

Supplementary Figure 6. Experimental results of ST-FMR measurements. a, Optical micrograph and the experimental setup of ST-FMR measurement. **b**, ST-FMR spectra of a 11 nm $SrRuO_3$/120 nm $BiFeO_3$/5 nm NiFe device measured at various frequencies. **c**, Device resistance as a function of magnetic field angle. The blue circles present the experimental data, the red curve represent the $sin2\varphi$ fitting curve. **d**, ST-FMR spectrum fitted to a Lorentzian function. The red and blue lines show the fits of the symmetric V_S and antisymmetric V_A components, respectively. **e**, Symmetric V_S and antisymmetric V_A components as a function of magnetic field angle φ at 7 GHz. The data is fitted to $sin2\varphi cos\varphi$. **f**, Resonance frequency as function of the resonance field (μ_0H_{res}), which is fitted to the Kittel equation.

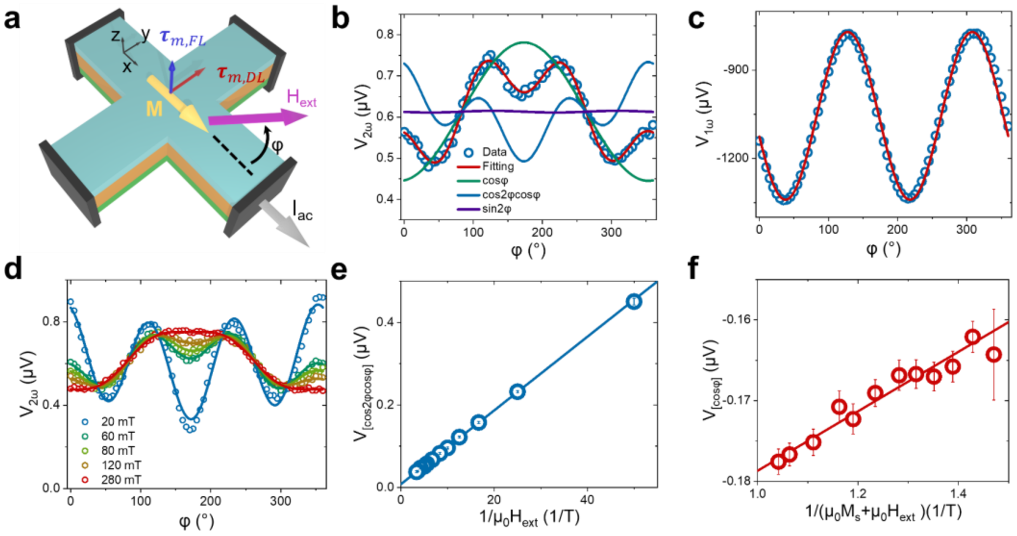

Supplementary Figure 7. Experimental results of SHHV measurements. a, Schematic diagram of the experimental setup for SHHV measurement. **b**, $V_{2\omega}$ as a function of the magnetic field angle φ with H_{ext} =80 mT for the 11 nm $SrRuO_3$/ 120 nm $BiFeO_3$/5nm NiFe. The circles represent the experimental data, and red curve represents the fit to Eq. (14). The green, blue and purple curves represent the fits of $cos\varphi$, $cos2\varphi cos\varphi$ and $sin2\varphi$ components, respectively. **c**, $V_{1\omega}$ as a function of the magnetic field angle φ with $\mu_0 H_{ext}$ = 80 mT for the same device. The circles present the experimental data, and the red curve represents the fitting curve to $cos2\varphi$. **d**, Second-harmonic Hall signals $V_{2\omega}$ as a function of the magnetic field angle φ with various field amplitudes for the same device. **e**, $cos2\varphi cos\varphi$-dependent $V_{2\omega}$ component plotted as a function of $1/H_{ext}$, where the error bars are due to the uncertainty of the fitting. **f**, $cos\varphi$-dependent $V_{2\omega}$ component plotted as a function of $\frac{1}{(\mu_0 M_s+\mu_0 H_{ext})}$, where the error bars are due to the uncertainty of the fitting.

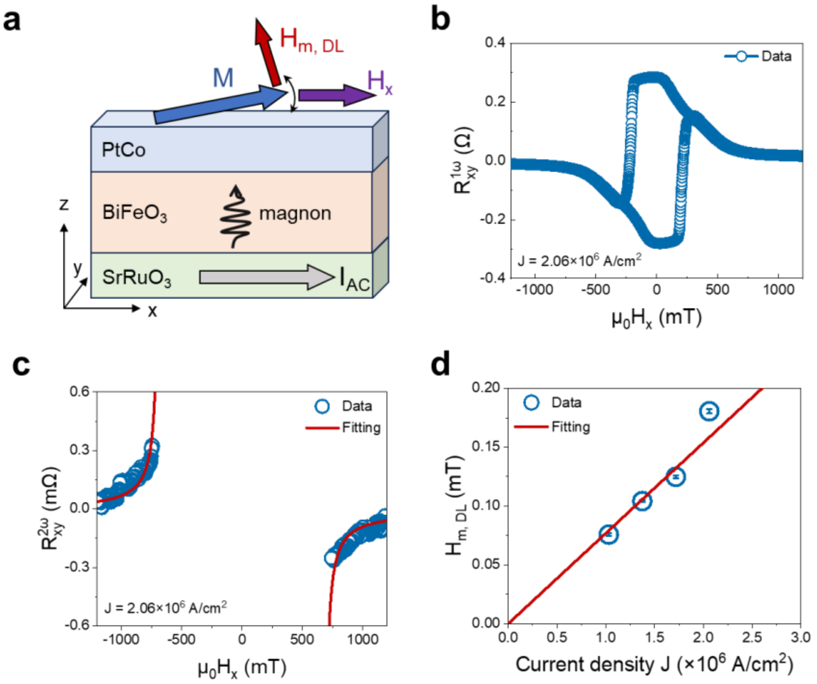

Supplementary Figure 8. The magnon-torque efficiency in a 11 nm $SrRuO_3$/120 nm $BiFeO_3$/PtCo sample. a, Schematic of second harmonic measurements. The magnetization M is oscillated by $H_{m,DL}$. The blue arrow indicates the direction of magnetization M. The experimental results of $R_{xy}^{1\omega}$ and $R_{xy}^{2\omega}$ are shown in **b** and **c**, respectively. The blue circles are the experimental data, and the red lines are the fitting curves. **d**, the effective field $H_{m,DL}$ as a function of current density. The blue circles are experimental results, and red line represents the linear fitting.

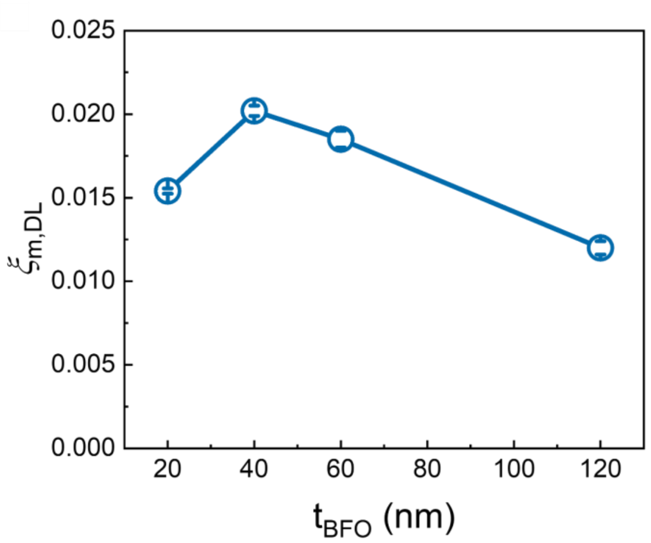

Supplementary Figure 9. Magnon torque efficiency $\xi_{m,DL}$ (measured by ST-FMR) of 11 nm $SrRuO_3/BiFeO_3$/5 nm NiFe samples with various $BiFeO_3$ thicknesses t_{BFO} = 20, 40, 60 and 120 nm.

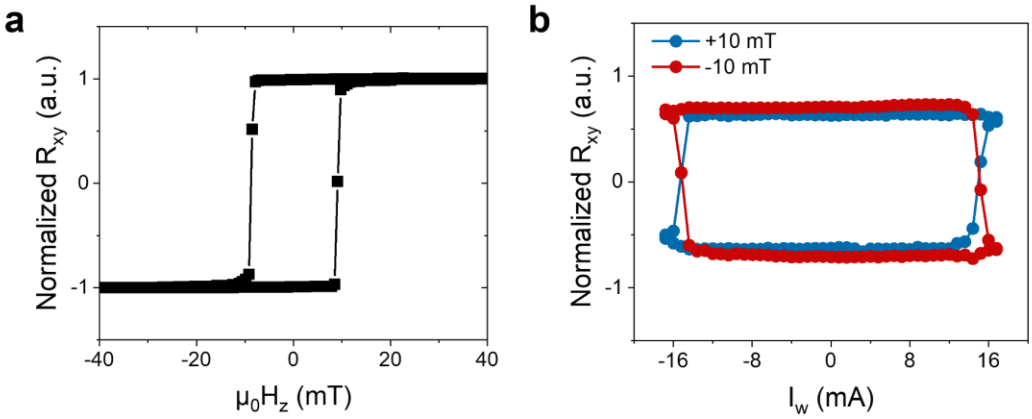

Supplementary Figure 10. Magnon-torque-induced switching in a 11 nm $SrRuO_3$/42 nm $BiFeO_3$/PtCo sample. a, Anomalous Hall resistance loop as sweeping out-of-plane magnetic fields. **b**, Magnon-torque-induced switching with the presence of an in-plane magnetic field $\mu_0H_x = \pm10$ mT.

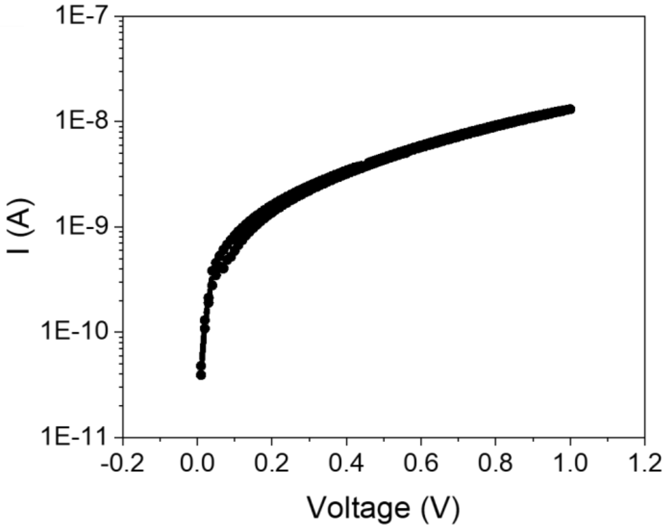

Supplementary Figure 11. Measured leakage current versus out-of-plane voltage in the device with 120 nm $BiFeO_3$/PtCo circular micro-pillars (25 µm diameter) positoned on a 11 nm $SrRuO_3$ channel.

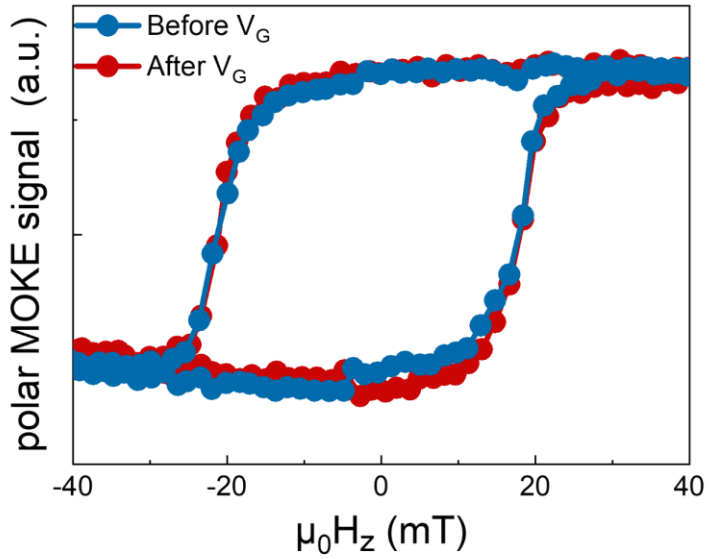

Supplementary Figure 12. Experimental results of polar MOKE measurements for the voltage-controlled magnetic anisotropy effect. Polar MOKE intensity obtained from the PtCo cell (as shown in Fig. 3) as a function of out-of-plane magnetic field H_z for the 11 nm $SrRuO_3$/120 nm $BiFeO_3$/PtCo sample before and after applying V_G.

	Logic function	Initial state (Out_{i-1})	Definition of IN
1	TRUE	1	1 ⇒ $-I_{c1}<I_w<I_{c1}$
		1	0 ⇒ $-I_{c1}<I_w<I_{c1}$
2	FALSE	0	1 ⇒ $-I_{c1}<I_w<I_{c1}$
		0	0 ⇒ $-I_{c1}<I_w<I_{c1}$
3	IN	1	1 ⇒ $-I_{c1}<I_w<I_{c1}$
		0	0 ⇒ $-I_{c1}<I_w<I_{c1}$
4	not IN	0	1 ⇒ $-I_{c1}<I_w<I_{c1}$
		1	0 ⇒ $-I_{c1}<I_w<I_{c1}$
5	W	1	1 ⇒ $-I_{c1}<I_w<I_{c1}$
		0	0 ⇒ $-I_{c1}<I_w<I_{c1}$
6	not W	0	1 ⇒ $-I_{c1}<I_w<I_{c1}$
		1	0 ⇒ $-I_{c1}<I_w<I_{c1}$
7	IN AND W	0	1 ⇒ $-I_{c2}<I_w<-I_{c1}$
		0	0 ⇒ $-I_{c1}<I_w<I_{c1}$
8	IN NAND W	1	1 ⇒ $I_{c1}<I_w<I_{c2}$
		1	0 ⇒ $-I_{c1}<I_w<I_{c1}$
9	IN OR W	1	1 ⇒ $-I_{c1}<I_w<I_{c1}$
		0	0 ⇒ $-I_{c2}<I_w<-I_{c1}$
10	IN NOR W	0	1 ⇒ $-I_{c1}<I_w<I_{c1}$
		1	0 ⇒ $I_{c1}<I_w<I_{c2}$
11	IN IMP W	0	1 ⇒ $-I_{c2}<I_w<-I_{c1}$
		1	0 ⇒ $-I_{c1}<I_w<I_{c1}$
12	IN NIMP W	1	1 ⇒ $I_{c1}<I_w<I_{c2}$
		0	0 ⇒ $-I_{c1}<I_w<I_{c1}$
13	IN RIMP W	1	1 ⇒ $-I_{c1}<I_w<I_{c1}$
		1	0 ⇒ $I_{c1}<I_w<I_{c2}$
14	IN RNIMP W	0	1 ⇒ $-I_{c1}<I_w<I_{c1}$
		0	0 ⇒ $-I_{c2}<I_w<-I_{c1}$
15	IN XOR W	1	1 ⇒ $I_{c1}<I_w<I_{c2}$
		0	0 ⇒ $-I_{c2}<I_w<-I_{c1}$
16	IN XNOR W	0	1 ⇒ $-I_{c2}<I_w<-I_{c1}$
		1	0 ⇒ $I_{c1}<I_w<I_{c2}$

Supplementary Figure 13. Definitions of IN and settings of OUT_{i-1} for performing all 16 Boolean logic functions. Different logic functions can be realized by setting the initial magnetization state (OUT_{i-1}) and configuring different amplitudes and polarities of I_w ($-I_{c2}<I_w<-I_{c1}$, $-I_{c1}<I_w<I_{c1}$ and $I_{c1}<I_w<I_{c2}$).

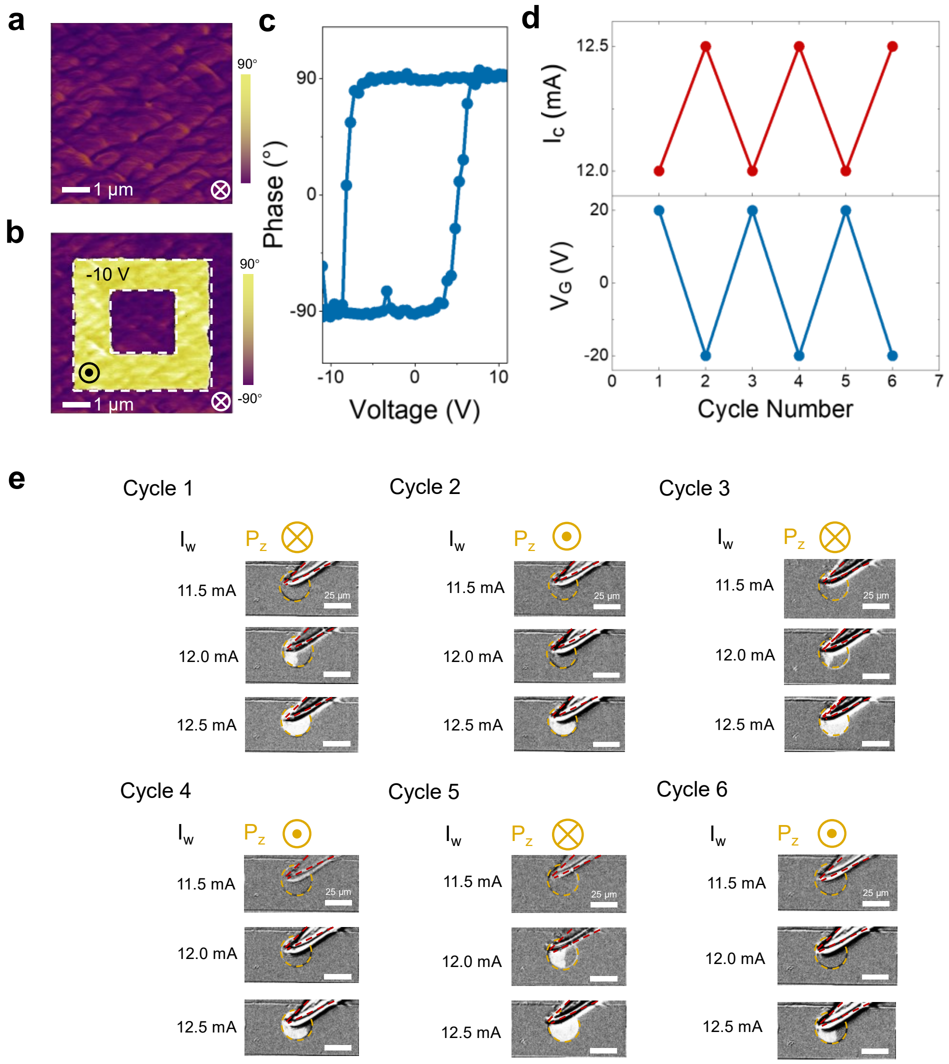

Supplementary Figure 14. Voltage control of magon-torque-induced switching in a sample with the mono-domain BiFeO₃. a-c, Ferroelectricity of the mono-domain $BiFeO_3$ in a 15 nm $SrRuO_3$/150 nm $BiFeO_3$. **a**, Out-of-plane PFM phase image of the as-grown sample. **b**, Out-of-plane PFM phase image written by bias voltages of -10 V. **c**, Out-of-plane PFM phase signal of PFM as a function of the applied voltage. **d**, Reversible modulation of the threshold current I_{th} for magnon-torque-induced switching after applying V_G for several cycles. **e**, Polar MOKE images of magnon-torque-induced switching for each cycle shown in **d**. The PtCo cells are denoted by yellow dash circles. Yellow ⊙|⊗ symbols indicate the direction of ferroelectric polarization of $BiFeO_3$. The red dash lines indicate the probe tip for applying V_G.

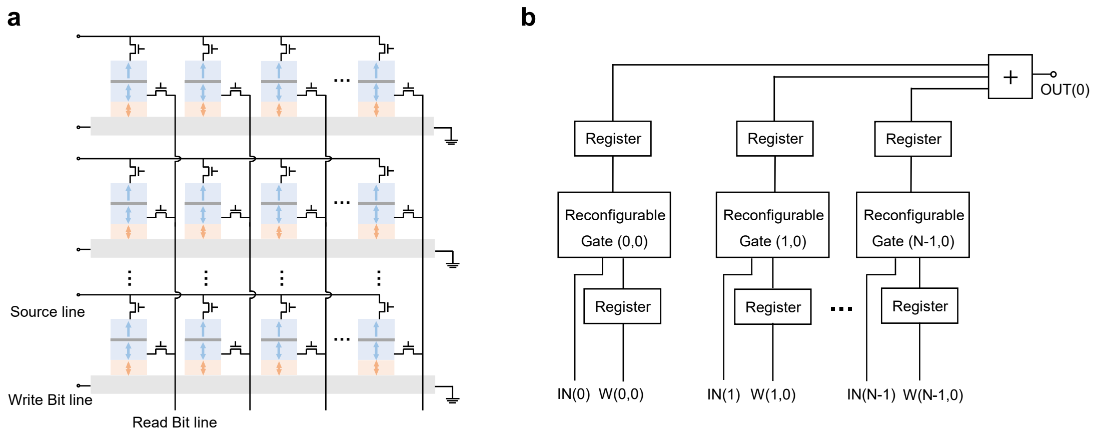

Supplementary Figure 15. MMST-MTJs crossbar array and the equivalent circuits. a, Schematic of MMST-MTJs crossbar array. **b**, Equivalent circuit of a MMST-MTJs column for the MAC operation.

Supplementary References

1 Qiu, Z. *et al.* Spin colossal magnetoresistance in an antiferromagnetic insulator. *Nat. Mater.* **17**, 577-580 (2018).

2 Rezende, S. M., Rodríguez-Suárez, R. L. & Azevedo, A. Diffusive magnonic spin transport in antiferromagnetic insulators. *Phys. Rev. B* **93**, 054412 (2016).

3 Bender, S. A., Skarsvag, H., Brataas, A. & Duine, R. A. Enhanced Spin Conductance of a Thin-Film Insulating Antiferromagnet. *Phys. Rev. Lett.* **119**, 056804 (2017).

4 Zheng, D. *et al.* High-efficiency magnon-mediated magnetization switching in all-oxide heterostructures with perpendicular magnetic anisotropy. *Adv. Mater.* **34**, 2203038 (2022).

5 Gross, I. *et al.* Real-space imaging of non-collinear antiferromagnetic order with a single-spin magnetometer. *Nature* **549**, 252-256 (2017).

6 Chu, Y. H. *et al.* Nanoscale domain control in multiferroic $BiFeO_3$ thin films. *Adv. Mater.* **18**, 2307-2311 (2006).

7 Liu, L. *et al.* Current-induced self-switching of perpendicular magnetization in CoPt single layer. *Nat. Commun.* **13**, 3539 (2022).

8 Nan, T. *et al.* Comparison of spin-orbit torques and spin pumping across NiFe/Pt and NiFe/Cu/Pt interfaces. *Phys. Rev. B* **91**, 214416 (2015).

9 MacNeill, D. *et al.* Control of spin-orbit torques through crystal symmetry in WTe_2/ferromagnet bilayers. *Nat. Phys.* **13**, 300-305 (2017).

10 Nan, T. *et al.* Anisotropic spin-orbit torque generation in epitaxial $SrIrO_3$ by symmetry design. *Proc. Natl. Acad. Sci. U.S.A.* **116**, 16186-16191 (2019).

11 Kang, M. G. *et al.* Electric-field control of field-free spin-orbit torque switching via laterally modulated Rashba effect in Pt/Co/AlO_x structures. *Nat. Commun.* **12**, 7111 (2021).

12 Avci, C. O. *et al.* Interplay of spin-orbit torque and thermoelectric effects in ferromagnet/normal-metal bilayers. *Phys. Rev. B* **90**, 224427 (2014).

13 Wu, H. *et al.* Room-Temperature Spin-Orbit Torque from Topological Surface States. *Phys. Rev. Lett.* **123**, 207205 (2019).

14 Zheng, Z. *et al.* Field-free spin-orbit torque-induced switching of perpendicular

magnetization in a ferrimagnetic layer with a vertical composition gradient. *Nat. Commun.* **12**, 4555 (2021).

15 Ishikuro, Y., Kawaguchi, M., Kato, N., Lau, Y.-C. & Hayashi, M. Dzyaloshinskii-Moriya interaction and spin-orbit torque at the Ir/Co interface. *Phys. Rev. B* **99**, 134421 (2019).

16 Wang, H. *et al.* Spin-orbit-torque switching mediated by an antiferromagnetic insulator. *Phys. Rev. Appl.* **11**, 044070 (2019).

17 Saenrang, W. *et al.* Deterministic and robust room-temperature exchange coupling in monodomain multiferroic $BiFeO_3$ heterostructures. *Nat. Commun.* **8**, 1583 (2017).

18 Haykal, A. *et al.* Antiferromagnetic textures in $BiFeO_3$ controlled by strain and electric field. *Nat. Commun.* **11**, 1704 (2020).

19 Qoutb, A. G. & Friedman, E. G. Double magnetic tunnel junction two bit memory and nonvolatile logic for in situ computing. *Microelectronics Journal* **131**, 105635 (2023).
